# Supplementary material for: Evaluation of the Dissemination of the South African 24-Hour Movement Guidelines for Birth to 5 Years
Source: Int J Environ Res Public Health. 2021 Mar 17;18(6):3071. doi: 10.3390/ijerph18063071 (PMC8002461; doi:10.3390/ijerph18063071)
Supplement: Supplementary file 1 [file ijerph-18-03071-s001.zip › Supplementary/ijerph-1111044-supplementary.docx]

**Group discussion guide questions**

- Do you work with ECD practitioners, or parents/caregivers of children from birth to five, or both? (If this has not already been clarified in the workshop)
- How do you think the workshop helped you to understand 24-hour movement behaviours?
- How important do you think the guidelines are for the health and development of 0-5-year-old children?
- Which of the recommendations in the guidelines do you think are most relevant in the setting where you work?
- Which of the recommendations in the guidelines do you think will be most difficult to implement in the setting where you work?
- What resources do you need to promote the guidelines with the people you work with?
- What support do you need to promote the guidelines with the people you work with?
- How do you think we could improve these workshops?
- Do you have any other comments you would like to make about the workshops?

**Follow-up focus group questions**

- Do you work with ECD practitioners, or parents/caregivers of children from birth to five, or both?
- Which of the recommendations in the guidelines do you think are most relevant in the setting where you work?
- Since the workshop last year, has anything changed about how important you think the guidelines are? (link to COVID-19 pandemic)
  - Physical health of 0-5-year-old children?
  - Mental development of 0-5-year-old children?
- How you found the process of sharing the guidelines with others?
- Which of the recommendations in the guidelines did you find the easiest to share?
- Which of the recommendations in the guidelines did you find the hardest to share?
- What resources do you use to share the guidelines?
  - Did you feel that you had sufficient resources?
  - If not, where do you think you could get these resources?
- What support did you have to share the guidelines?
  - What additional support did you need / do you need going forward?
  - How do you think you could support others to promote the guidelines?
- How confident do you feel that you can continue to share these guidelines with the people you work with?

*[Explain Woza, Mntwana song]*

- What do you think of the idea to share the guidelines messages through music?
- How do you think South Africans would respond?
- Who do you think would be our ideal target market?

*[Play Woza, Mntwana song]*

- What do you think of the song?
- How do you think we can best share this song to reach the most people in South Africa?
